# Biochemical and Biophysical Properties of Interactions between Subunits of the Peripheral Stalk Region of Human V-ATPase

**DOI:** 10.1371/journal.pone.0055704

**Published:** 2013-02-11

**Authors:** Suhaila Rahman, Ichiro Yamato, Shinya Saijo, Kenji Mizutani, Yoshiko Ishizuka-Katsura, Noboru Ohsawa, Takaho Terada, Mikako Shirouzu, Shigeyuki Yokoyama, So Iwata, Takeshi Murata

**Affiliations:** 1 Department of Biological Science and Technology, Tokyo University of Science, Chiba, Japan; 2 RIKEN SPring-8 Center, Hyogo, Japan; 3 Department of Chemistry, Graduate School of Science, Chiba University, Chiba, Japan; 4 RIKEN Systems and Structural Biology Center, Yokohama, Japan; 5 Department of Biophysics and Biochemistry, Graduate School of Science, The University of Tokyo, Tokyo, Japan; 6 Department of Cell Biology, Faculty of Medicine, Kyoto University, Kyoto, Japan; 7 JST, PRESTO, Chiba, Japan; Centro Nacional de Biotecnologia - CSIC, Spain

## Abstract

Peripheral stalk subunits of eukaryotic or mammalian vacuolar ATPases (V-ATPases) play key roles in regulating its assembly and disassembly. In a previous study, we purified several subunits and their isoforms of the peripheral stalk region of *Homo sapiens* (human) V-ATPase; such as C1, E1G1, H, and the N-terminal cytoplasmic region of V_o_, a1. Here, we investigated the *in vitro* binding interactions of the subunits at the stalk region and measured their specific affinities. Surface plasmon resonance experiments revealed that the subunit C1 binds the E1G1 heterodimer with both high and low affinities (2.8 nM and 1.9 µM, respectively). In addition, an E1G1-H complex can be formed with high affinity (48 nM), whereas affinities of other subunit pairs appeared to be low (∼0.21−3.0 µM). The putative ternary complex of C1-H-E1G1 was not much strong on co-incubation of these subunits, indicating that the two strong complexes of C1-E1G1 and H-E1G1 in cooperation with many other weak interactions may be sufficiently strong enough to withstand the torque of rotation during catalysis. We observed a partially stable quaternary complex (consisting of E1G1, C1, a1_NT_, and H subunits) resulting from discrete peripheral subunit interactions stabilizing the complex through their intrinsic affinities. No binding was observed in the absence of E1G1 (using only H, C1, and a1_NT_); therefore, it is likely that, *in vivo*, the E1G1 heterodimer has a significant role in the initiation of subunit assembly. Multiple interactions of variable affinity in the stalk region may be important to the mechanism of reversible dissociation of the intact V-ATPase.

## Introduction

**Figure 1:**
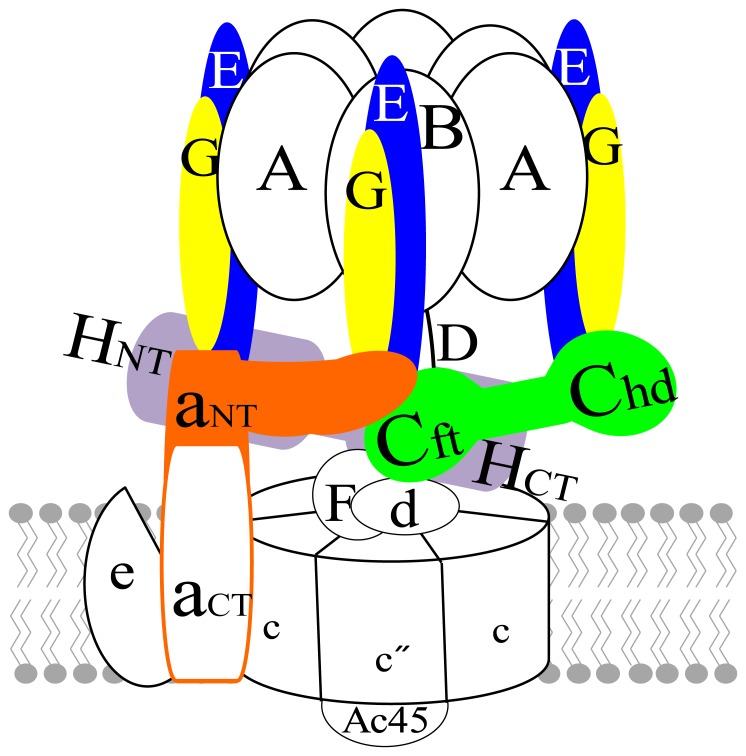
Structural model of human V-ATPase. Peripheral stalk subunits (E, G, C, a_NT_, and H) are emphasized using colors in the schematic model.

In mammals, vacuolar ATPases (V-ATPases) are found within endomembrane systems where their ATP hydrolysis-driven proton pump function has important roles in crucial cellular processes. Their malfunction can, therefore, cause diverse pathophysiological states in humans [1]–[6]. Eukaryotic V-ATPases are multiprotein complexes consisting of at least 14 different polypeptide chains. The sophisticated complex has a structure consisting of membrane-integrated V_o_ and cytoplasmic extrinsic V_1_ domains. V_o_ and V_1_ domains are linked by a connecting region consisting of a central shaft and multiple peripheral stator elements. The V_1_ domain contains three copies each of A, B, E, and G subunits and one copy each of C, D, F, and H subunits (Figure 1). It has a head group consisting of three nucleotide-binding A-B dimers responsible for ATP hydrolysis and two peripheral and central stalks consisting of the remaining V_1_ subunits, with distinct functions in the rotary mechanism by which the V-ATPase couples ATP hydrolysis to proton transport. The central stalk serves as a shaft that couples the energy of ATP hydrolysis to the rotation of a ring of proteolipid subunits in V_o_. Conversely, the peripheral stalks serves to prevent rotation of the A_3_B_3_ head during ATP hydrolysis and acts as a structural link between V_1_ and V_o_, thus providing physical support for the whole complex.

Crosslinking and electron microscopy studies have provided evidence that C, E, G, H, and the N-terminal domain of the a subunit (a_NT_) all form part of the peripheral stalk [1], [7], [8]. C and H are the two major subunits without functional homologues in either the F- or A-type ATPases, or bacterial A/V-ATPases. In eukaryotes/mammals, three peripheral stalks, consisting of three pairs of EG heterodimers along with C, H, and a_NT_, function to resist the torque of rotation by supporting an A_3_B_3_ hexamer during catalysis. Two pairs of EG interact with the C subunit via its foot (C_foot_) and head (C_head_) domains, each interaction has different affinities, and the third pair of EG interacts with the H subunit [9], [10], [11]. The C subunit is known to play a critical role in connecting V_1_ and V_o_ by linking one peripheral stator EG heterodimer via the C_head_ region to a ternary junction point where C_foot_, a_NT_, and another peripheral stator EG are also involved in the interaction [8], [9], [10], [12], [13] (Figure 1). Recently, Oot and Wilkens proposed a model of this ternary junction point in yeast V-ATPase, consisting of C_foot_, EG, and a_NT_ subunits, from experiments using a truncated a_NT_ domain (104–372 amino acid residues) and the C_foot_ region (residues 1–151 and 287–392) of the C subunit [14], [9]. They proposed that the interactions in this ternary junction point are of high avidity and enable the V_1_ and V_o_ domains to resist the torque of rotational catalysis. Furthermore, the differences in the interaction affinities of the C subunit with E, G, H, and a_NT_ are potentially essential for reversible dissociation, in which the C subunit completely dissociates from the V_1_–V_o_ complex. Studies of eukaryotic V-ATPases have led to the proposal that this reversible dissociation mechanism is common to yeast, insects, and mammals [2], [15]–[21]. However, the majority of biochemical and biophysical studies of subunit interactions have been performed in yeast. The lack of data regarding mammalian subunit-subunit interactions necessitates ascertainment of the binding phenomena in a mammalian system. Sequence similarity between yeast and human subunits is relatively low (identity 31%−41% and similarity 51%−60%, depending on subunits and isoforms); hence, it may be questionable to assume that their structural organization and biochemical properties are alike. Therefore, a detailed biochemical and structural understanding of the topological arrangement of a mammalian V-ATPase is necessary to unravel the mechanistic relationship between regulation and activity of the V_1_ domain in mammals. We expressed and purified several isoforms of the human peripheral stalk subunits, utilizing a cell-free protein expression system [22]. In this study, we performed binding interaction studies utilizing one representative isoform of each subunit (E1G1, C1, N-terminal domain of a1 isoform of human V-ATPase (a1_NT_) and H) to determine the binding phenomena of these subunits in stator structure and function. Subunit E1 and G1 alone were unstable and could not be expressed. Therefore, we tried co-expression and co-purification of the E1G1 complex [22]. We measured binary interaction affinities between the subunits and characterized ternary and quaternary complexes. This report of the peripheral region subunit-subunit interaction properties of a mammalian V-ATPase facilitates understanding of the properties and mechanisms of regulation of this most complex enzyme.

## Materials and Methods

### Human Protein Expression and Purification

Human V-ATPase protein subunits (C1, H, E1G1, and a1_NT_) were expressed using an *Escherichia coli* cell-free protein synthesis system and purified as previously described [22]. Purified proteins were stored at −80°C until use.

### Purification of His-tagged H Subunit

The His-tagged H subunit was purified by loading the cell-free synthesizing mixture onto a 5 ml HisTrap HP column (GE Healthcare, Buckinghamshire, England) equilibrated with buffer B (20 mM Tris-HCl pH 8.0, 1 M NaCl, 20 mM imidazole). Bound proteins eluted with buffer C (20 mM Tris-HCl pH 8.0, 0.5 M NaCl, 500 mM imidazole). Proteins were dialyzed against buffer H (20 mM Tris-HCl pH 8.5, 20 mM NaCl, 2 mM Dithiothreitol (DTT) at 4°C overnight. The sample was applied to an ion exchange column (HiTrap Q, 5 ml; GE Healthcare, Buckinghamshire, England), and all further purification was performed as described for the H subunit [22]. His-tagged H subunit protein eluted as a single peak (estimated MW = 59,294 Da and PI = 6.3).

### Gel Filtration Chromatography

The purified proteins of human E1G1, C1, H, and a1_NT_ were loaded individually on a Superdex 200 (5/150) column (GE Healthcare) connected to a BioLogic Duo-Flow system (Bio-Rad). Protein-protein binding interactions of different combinations of these proteins were analyzed by incubating mixtures of varying molar ratios of the purified proteins on ice for approximately 1 h and then passing the mixture through the column. Complexes formed by protein-protein interactions were identified by shifts of peak position and confirmed by analysis of the eluted fractions by sodium dodecyl sulfate-polyacrylamide gel electrophoresis (SDS-PAGE). Gel filtration was performed at 4°C, in 20 mM 2-[4-(2-hydroxyethyl)-1-piperazinyl] ethanesulfonic acid (HEPES)-NaOH, 300 mM NaCl, 1 mM DTT, pH 7.5. The absorbance of the column eluate (fraction volume, 0.05 ml) was monitored at 280 nm. The column was calibrated using blue dextran, ferritin (440 kDa), aldolase (158 kDa), conalbumin (75 kDa), and ovalbumin (44 kDa).

### Native PAGE

Basic native gel electrophoresis was carried out according to an online protocol (http://wolfson.huji.ac.il/purification/Protocols/PAGE_Basic.html). For analysis of the binding interactions, different molar ratios of protein subunits were mixed and incubated on ice for approximately 1 h. Gel electrophoresis was carried out at 4°C for approximately 4 h, and the gel was stained with Coomassie Brilliant Blue R250. Native complexes were analyzed in a second denaturing dimension. Bands corresponding to the respective complexes were excised from the native gel and incubated in 30 µl of SDS sample buffer for overnight at 4°C for the elution of proteins. The eluted solutions from the excised band were then loaded on the denaturing gel and SDS-PAGE was performed according to the method described by Laemmli [23].

### Pulldown Assay Using His-tagged H Subunit

His-tagged V_1_-H protein was mixed with non-tagged samples and incubated for 1 h on ice. The mixture was incubated with Ni Sepharose 6 Fast Flow pre-equilibrated with buffer A (20 mM HEPES-NaOH, 300 mM NaCl, 0.1 mM DTT, pH 7.5) at 4°C in a rotator for 1 h and washed thrice with 5 column volumes of buffer A. Loosely bound proteins were eluted using buffer B (20 mM HEPES-NaOH, 300 mM NaCl, 0.1 mM DTT, 10 mM imidazole, pH 7.5) by washing thrice with 5 column volumes. Finally, bound proteins were eluted using buffer C (20 mM HEPES-NaOH, 300 mM NaCl, 0.1 mM DTT, 500 mM imidazole, pH 7.5). All eluted proteins were analyzed by SDS-PAGE.

### Surface Plasmon Resonance (SPR) Analysis for Real-time Binding Assay

Purified human proteins (30 µg/ml) C1, H, and a1_NT_ in 10 mM sodium acetate buffer at pH 5.0, 4.5, and 4.25, respectively, were separately immobilized on a CM5 sensor chip using an amine coupling kit according to the manufacturer’s instructions. The running buffer used contained 20 mM HEPES-NaOH, 300 mM NaCl, 1 mM DTT, 0.05% surfactant P20 (Tween 20), pH 7.5. The purified sample was used as the analyte and was passed over the surface of the sensor chip with a typical flow rate of 10 µl/min at 10°C, and the interactions were monitored. The sensor surface was then washed with a regeneration solution (0.1% sodium lauroyl sarcosinate (Sarkosyl) in 20 mM HEPES-NaOH, 150 mM NaCl, pH 7.5) for 30 s [24]. For determining the binding interactions of the C1-E1G1 complex, the association and dissociation rate constants (*k*_a_ and *k*_d_) were calculated using BIAevaluation software (Version 1.1, GE Healthcare, Uppsala, Sweden) and the heterogeneous ligand binding model. The dissociation constant (*K_D_*) was calculated from the *k*_d_/*k*_a_ values. For other subunit-subunit interactions, it was not possible to obtain the rate constants because of low affinity and inadequate curve fitting; therefore, we obtained *K_D_* from the plots of steady-state analyte binding levels (*R*_eq_) against several analyte concentrations at equilibrium. The fitted curve (*R*_eq_ *versus* concentrations of analyte) and *K_D_* values were obtained using the BIAevaluation software with a single 1∶1 (Langmuir) binding isotherm.

### Others

Densitometric analysis of SDS-PAGE gels was performed using the ImageJ software (National Institutes of Health, Bethesda, Maryland, USA). All reagents used for this study were of analytical grade and obtained from Sigma-Aldrich (K. K., Tokyo, Japan), Wako Pure Chem. (Tokyo, Japan), or Dojindo Chemicals (Tokyo, Japan). The Biacore T100 instrument system and reagents, including sensor chip (CM5) and the amine coupling kit, were obtained from GE Healthcare (Uppsala, Sweden).

## Results

### Stable Binding Interaction in E1G1-C1 and E1G1-H Complexes

**Figure 2:**
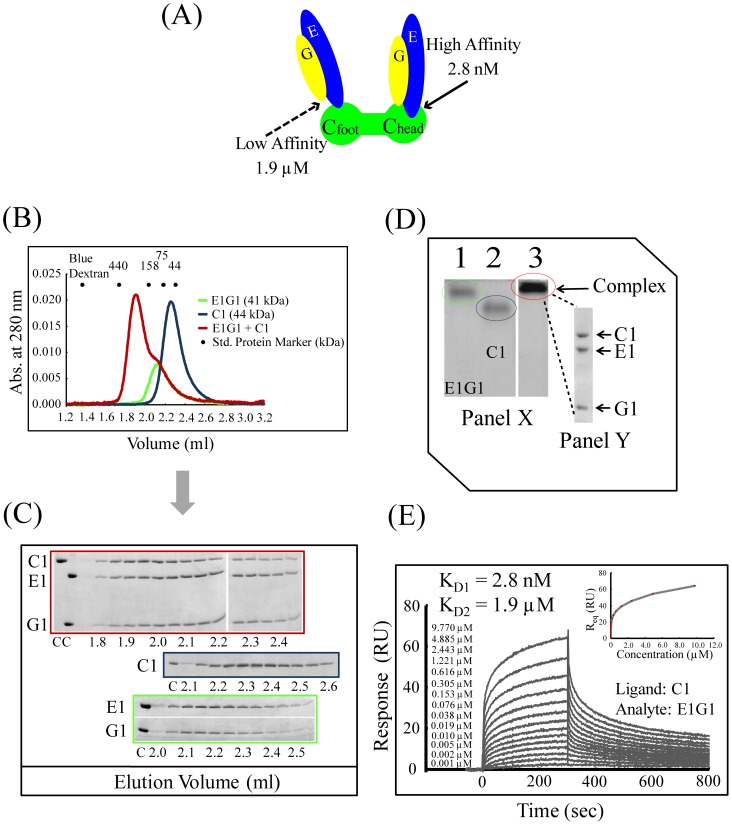
Interactions between E1G1 and C1. (A) Mode of E1G1-C1 binding interaction *in vitro* based on data reported from previous studies of yeast [11]. Using a Biacore system, the *K_D_* values for affinities of C1_head_-E1G1 and C1_foot_-E1G1 were estimated to be 2.8 nM and 1.9 µM, respectively, as shown in 2D. (B) Gel filtration profile of E1G1/C1 complex formation (red) in comparison with E1G1 (green) and C1 (blue) monomers. (C) SDS-PAGE analysis of the eluted fractions from gel filtration chromatography. Gel border colors indicate samples corresponding to the color scheme used in 2B. “C” indicates control proteins. (D) Panel X: Basic native polyacrylamide gel electrophoresis analysis of E1G1 and C1 interaction. For complex formation, a 2∶1 molar ratio of E1G1:C1 proteins was prepared and incubated on ice for 1 h (lane 3). Bands corresponding to one molar amount of E1G1 and C1 are visible in lanes 1 and 2, respectively. Panel Y: SDS-PAGE (12% gel) analysis of the E1G1C1 complex band eluted from the native gel in panel X (lane 3). (E) Real-time binding evaluation was performed using a Biacore system. Sensorgrams for the binding of various concentrations of the analyte (E1G1) to the ligand (C1) are shown. The inset curve shows the steady-state binding isotherm for binding of E1G1 at various concentrations to C1 ligand on a CM5 sensor chip.

Subunit-subunit interactions were examined by several methods to determine genuine binding between E1G1 (a representative of all EG subunits) and both C1 (a representative of C subunits) and H subunits. Previous studies in yeast have shown that the C subunit can interact with two EG subunit pairs with different affinities [8], [11] (Figure 2A). By mixing E1G1 and C1 in a 2∶1 molar ratio, the E1G1C1 complex eluted as a peak with a small subsequent shoulder earlier than the corresponding monomers (E1G1 complex or C1 monomer) from the gel filtration column (Figure 2B); since E1 and G1 alone were unstable, they were co-expressed and co-purified as E1G1 complex [22]. The eluted fractions were analyzed by SDS-PAGE, and the complex formation was confirmed by the appearance of the C1 and E1G1 complex bands in fractions eluted earlier than their monomers (Figure 2C). The shoulder peak contained small amount of unbound C1 and excess E1G1, which was visible in the later fractions as analyzed by SDS-PAGE. Densitometric analysis of SDS-PAGE gels was performed using the ImageJ software to compare both equimolar E1G1-C1 (data not shown) and a 2∶1 molar ratio of E1G1:C1. Using the staining intensity of C1, E1, and G1 bands (Figure 2C) and estimating protein amounts using standard curves determined by us (Figure S1), the binding stoichiometry of C1 vs. E1G1 was found to be 1∶1, suggesting that the low-affinity binding site of the C1-foot (C1_foot_) region [11] failed to interact with E1G1. The molecular size of the complex estimated (on the basis of the calibration curve of standard proteins) from the eluted position was approximately 220 kDa, which is much higher than the calculated molecular weight (41+44 = 85 kDa). There are two possible explanations for this early elution: Either dimer formation of the C1-E1G1 complex (85×2 = 170 kDa) or the L-shaped structure of this complex [10], [25]. We did not have any other strong evidence for the dimerization of this complex; hence, the inconsistency probably was due to the effect of the L-shape of the complex, which migrates in a different manner from globular-shaped proteins.

**Figure 3:**
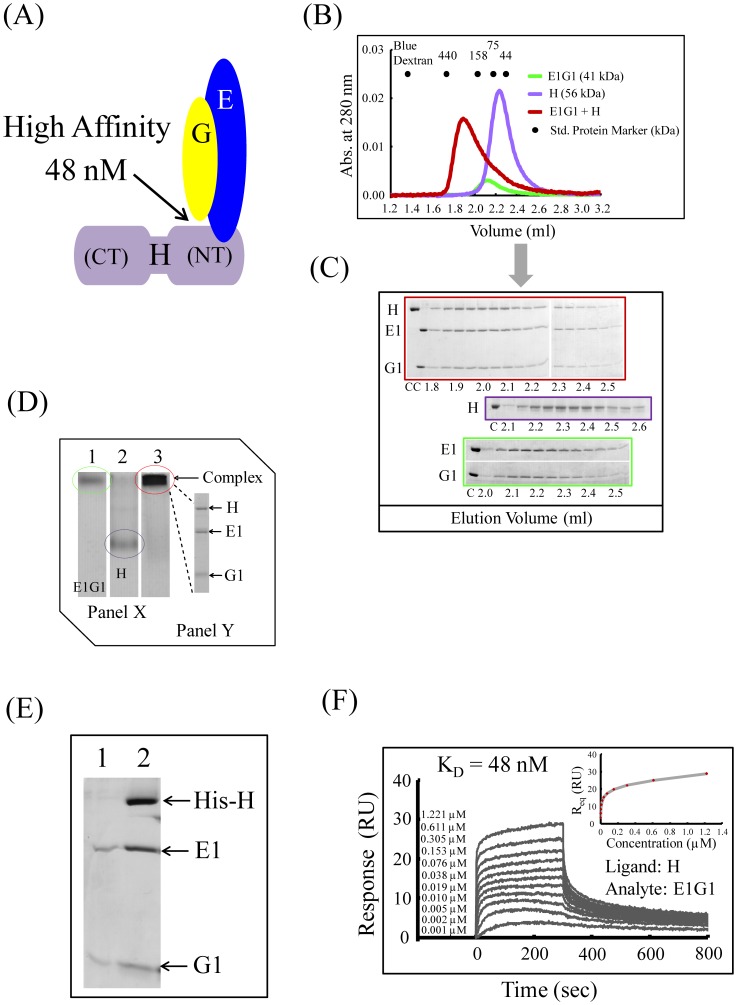
Interactions between E1G1 and H. (A) Possible mode of E1G1-H binding interaction *in vitro*. Using a Biacore system, the *K_D_* values for affinity of H-E1G1 was estimated to be 48 nM. (B) Gel filtration profile of E1G1/H complex formation (red) in comparison to E1G1 (green) and H (purple) monomers. (C) SDS-PAGE analysis of the eluted fractions from gel filtration chromatography. Gel Border colors indicate samples corresponding to the color scheme used in 3B. “C” indicates control proteins. (D) Panel X: Basic native polyacrylamide gel electrophoresis analysis of E1G1 and H interaction. For complex formation, equimolar amounts of E1G1 and H proteins were mixed and incubated on ice for 1 h (lane 3). Bands corresponding to one molar amount of E1G1 and H are visible in lanes 1 and 2, respectively. Panel Y: SDS-PAGE (12% gel) analysis of the E1G1H complex band eluted from the native gel in panel X (lane 3). (E) SDS-PAGE of the eluted proteins from the His-tag pulldown experiment. Lane 1, fractions eluted using buffer B; lane 2, E1G1 complex bound with His-tagged H subunit eluted using buffer C. (F) Real-time binding evaluation was performed using a Biacore system. Sensorgrams for the binding of various concentrations of the analyte (E1G1) to the ligand (H) are shown. The inset curve shows the steady-state binding isotherm for binding of E1G1 at various concentrations to H ligand on a CM5 sensor chip.

We observed similarly stable complex formation of E1G1 and H subunits (Figure 3A). Again, the equimolar complex of E1G1 and H eluted earlier with an apparent molecular weight of approximately 220 kDa, which is much higher than that calculated (Figure 3B). Complex formation was confirmed by analyzing the fractions by SDS-PAGE. Densitometric analysis of the gel filtration fractions of E1G1-H indicated that the binding stoichiometry of H vs. E1G1 was 1∶1.2 (Figure 3C and Figure S1). The anomalous early elution of these stator subunits alone (especially E1G1) or as a complex (E1G1-C1 or E1G1-H) can be interpreted as the effect of the elongated structure of these subunits, and is consistent with previous finding in studies on yeast [10], [25], [26].

To confirm the high affinity interactions of E1G1-C1 and E1G1-H, electrophoretic mobility shift assays on polyacrylamide gels were performed under native conditions. Figure 2D and Figure 3D show the native gel profiles for E1G1-C1 and E1G1-H complex formation, respectively. Subunit E1 (pI ∼8.5) and G1 (pI ∼9.3) have basic pI values (pI values were predicted from the protein sequence); however, the resultant E1G1 could be detected by basic native PAGE (Figure 2D and 3D, lane 1). C1 (pI ∼7.3) and H (pI ∼6.0) have acidic pIs; thus, they were clearly separated by basic PAGE (Figure 2D and 3D, lane 2). E1G1-C1 and E1G1-H complexes showed limited migration (Figure 2D and 3D, lane 3). The E1G1-C1 and E1G1-H complexes were excised from the native gel and analyzed by SDS-PAGE (Figure 2D and Figure 3D, Panel Y). The results by electrophoretic mobility shift assay are consistent with E1G1-C1 and E1G1-H complex formation. His-tagged H subunit was used to further confirm the interaction with E1G1 by a pulldown assay on Ni Sepharose 6 Fast Flow (Figure 3E, lane 2).

Surface plasmon resonance (SPR) analysis was performed for the quantitative estimation of the binding affinities of the subunit-subunit interactions, E1G1 with C1 and E1G1 with H (Figure 2E and 3F). Subunit C1 or H was immobilized as ligand on CM5 chip, and specific binding responses were observed when various concentrations of E1G1 were flowed over the immobilized sensor chips. It was possible to determine two binding affinities (with *K_D_* values of 2.8 nM and 1.9 µM) of subunit C1 for E1G1 using the BIAevaluation software with a heterogeneous ligand binding model; these were assumed to be affinities between C1_head_ and E1G1, and C1_foot_ and E1G1, respectively, as per previous studies in yeast [11]. Since the SPR on-rate and off-rate appear to be fast in the binding of E1G1 to H, it was difficult to calculate the kinetic parameters (*k*_on_ and *k*_off_) directly. Therefore, the affinity of the interaction was estimated from the level of binding at equilibrium as a function of the sample concentrations; a *K_D_* value of 48 nM for the E1G1-H interaction was obtained.

### Weak Binding Interactions between C1-H, E1G1-a1_NT_, C1-a1_NT_, and H-a1_NT_

We examined moderate-to-low binding interactions between other subunits (C1-H, E1G1-a1_NT_, C1-a1_NT_, and H-a1_NT_) of the peripheral stalk by using the methods described above. Strong binding and stable complex formation between these subunits was not observed in native gel electrophoresis or gel filtration. Pulldown assays using His-tagged protein could not identify a clear binding interaction of C1-H (Figure S2E, lane 2) and H-a1_NT_ (Figure S5D, lane 2). To measure the binding affinities and kinetics of (C1-H), (E1G1-a1_NT_), (C1-a1_NT_), and (H-a1_NT_) complexes, quantitative estimations were performed using a Biacore system. We were unable to estimate the kinetic parameters in all cases because of unreliable curve fitting of these low affinity binding interactions. Moreover, experiments could not be performed using very high concentrations of analytes to avoid the influence of non-specific background responses. We obtained similar affinity constants from the binding interactions of these subunits even when the ligand and analyte positions were reversed in the quantitative analysis (N.B. E1G1 was never used as a ligand because it is a heterodimer). Finally, for the estimation of other binary binding affinities, we calculated *K_D_* from the plots of steady-state analyte binding levels (*R*_eq_) against several analyte concentrations at equilibrium. The *K_D_* values are summarized in Table 1. These micro-molar order affinities indicate weak binding interactions, which may be important for the dissociation of the interaction during enzyme regulation (Figures S2, S3, S4, S5).

**Table 1:** Summary of the Biacore experiment results for subunit-subunit binding interactions.

Ligand(Molecular mass)		Analyte	Association rate	Dissociation rate	Affinity
			*k_a_* (M^−1^s^−1^)	*k_d_* (s^−1^)	*K_D_* (M)
					
C1 (44,460)		E1G1	3.7×10^6^	0.011	2.8×10^−9^
C1 (44,460)		E1G1	5.4×10^2^	0.01	1.9×10^−6^
H (56,402)		E1G1	……….	……….	4.8×10^−8^
H (56,402)		C1	……….	……….	1.4×10^−6^
C1 (44,460)		a1_NT_	……….	……….	3.0×10^−6^
a1_NT_ (41,532)		E1G1	……….	……….	2.1×10^−7^
H (56,402)		a1_NT_	……….	……….	3.6×10^−7^

### Investigation of Ternary Interaction in an E1G1-C1-H Mixture

**Figure 4:**
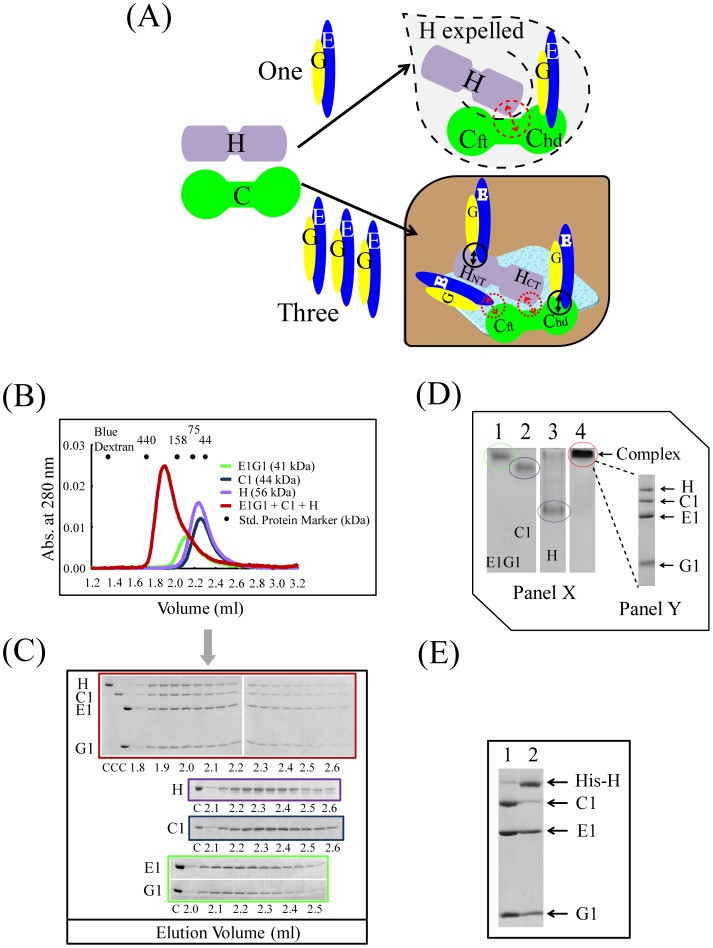
Ternary interactions of E1G1, C1, and H. (A) Model of E1G1-C1-H assembly. Dotted arrows (red) and solid arrows (black) indicate weak and strong binding, respectively. (B) Gel filtration profile of the H/C1/E1G1 mixture (red) in comparison to H (purple), E1G1 (green), and C1 (blue) monomers. (C) SDS-PAGE analysis of the eluted fractions from gel filtration chromatography. Border colors indicate samples corresponding to the color scheme used in 4B. “C” indicates control proteins. (D) Panel X: Basic native polyacrylamide gel electrophoresis analysis of the H/C1/E1G1 mixture. A molar ratio of 3∶1∶1 of E1G1:C1:H proteins was prepared and incubated on ice for 1 h (lane 4). Bands corresponding to one molar amount of E1G1, C1, and H proteins are visible in lanes 1, 2, and 3, respectively. Panel Y: SDS-PAGE (12% gel) analysis of the E1G1-C1-H mixture band eluted from the native gel in panel X (lane 4). (E) SDS-PAGE of the eluted proteins from the His-tag pulldown experiment. Lane 1, fraction eluted using buffer B; lane 2, subunits bound with His-tagged H subunit eluted using buffer C.

To determine higher-order binding among subunits, ternary and quaternary binding interactions were examined. Interestingly, complex formation was not observed using equimolar amounts of E1G1, C1, and H, with the H subunit being expelled, appearing as two peaks in the gel filtration analysis (–B) (model shown in Figure 4A). We expected that by using a 3∶1∶1 molar ratio of E1G1:C1:H, a stable CE_3_G_3_H putative ternary complex would be formed. Unexpectedly, however, in gel filtration, this mixture of proteins appeared as single peak at 1.85 ml (Figure 4B–C), a position similar to that of C1-E1G1 and H-E1G1 complexes (Figure 2B–C and Figure 3B–C). The binding of one E1G1 with the C1_foot_ region was not sufficiently strong for it to remain in the complex to form CE_3_G_3_H; instead it remained unbound, leading to the formation of a C1(E1G1)_2_H complex. We interpreted that the E1G1-C1-H putative ternary complex was not formed because of the lower binding affinities between C1-H and E1G1-C1_foot_; thus, we thought that the peak in gel filtration contained a mixture of two strong complexes (E1G1-C1_head_ and E1G1-H). The integrity of the putative ternary complex (E1G1-C1-H) formation was further examined by basic native PAGE, by mixing a similar molar ratio of each protein as for gel filtration (Figure 4D). In comparison to subunit C1, E1G1, or H, the migration rate of the (E1G1-C1-H) complex was delayed. The band corresponding to the complex was excised from the native gel and analyzed by SDS-PAGE (Figure 4D; Panel Y), with results consistent with the existence of a mixture of E1G1-C1 and E1G1-H complexes. A pulldown assay using His-tagged-H subunit showed strong binding with E1G1 but weak binding of C1, suggesting no stable putative ternary complex was formed (Figure 4E, lane 2). We speculate that two binary complexes (E1G1C1 and E1G1H) are sufficiently strong, together with other weak binding interactions, to form the L-shaped structure of CE_3_G_3_H *in vivo* [27].

### Investigation of Ternary Interactions in an E1G1-a1_NT_-C1 Mixture

**Figure 5:**
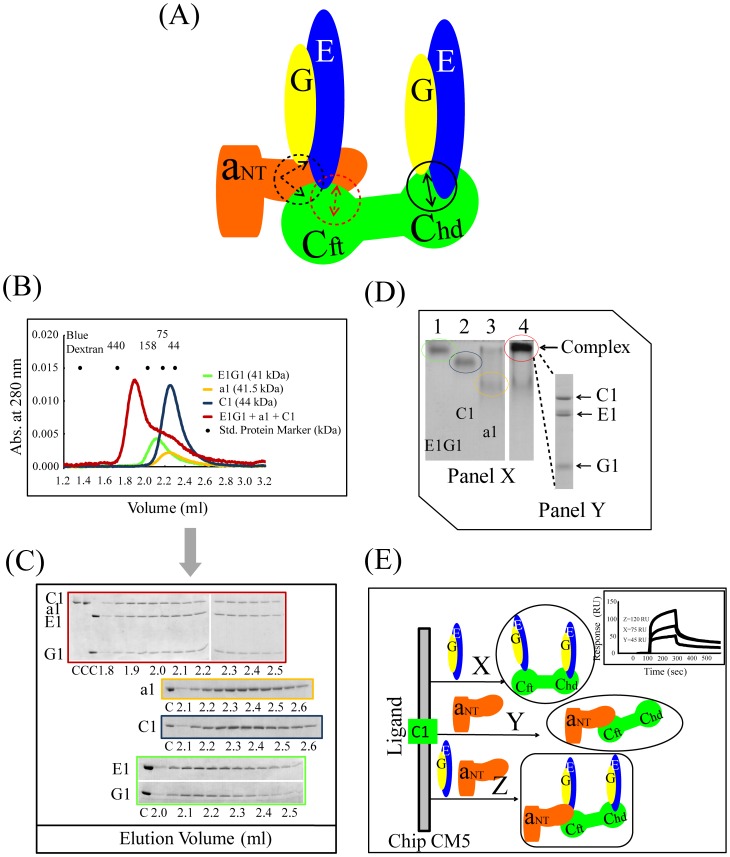
Ternary interactions of E1G1, C1, and a1_NT_. (A) Possible model of ternary binding interaction of E1G1, C1 and a1_NT_. Dotted arrows indicate weak and solid arrows (black) strong binding. (B) Gel filtration profile of E1G1/C1/a1_NT_ mixture (red) in comparison to E1G1 (green), C1 (blue) and a1_NT_ (yellow) monomers. (C) SDS-PAGE analysis of the eluted fractions from gel filtration chromatography. Border colors indicate samples corresponding to the color scheme used in 5B. “C” indicates control proteins. (D) Panel X: Basic native polyacrylamide gel electrophoresis analysis of the E1G1/C1/a1_NT_ mixture. A 2∶1∶2 molar ratio of E1G1:C1:a1_NT_ proteins was prepared and incubated on ice for 1 h (lane 4). Bands corresponding to one molar amount of E1G1, C1 and a1_NT_ proteins are visible in lanes 1, 2, and 3, respectively. Panel Y: SDS-PAGE (12% gel) analysis of the strong band eluted from the native gel in panel X (lane 4), suggesting the presence of C1, E1, and G1 in the complex. An unbound a1_NT_ band was observed at the expected position. (E) Model showing the binding mode interpreted from the Biacore data (inset) where ligand was C1: X, the binding model of E1G1 as analyte (10 µM protein). Y, the binding model of a1_NT_ as analyte (10 µM protein). Z, the binding model of E1G1 and a1_NT_ as analytes (10 µM of each protein). The inset shows the sensorgrams of X, Y, and Z binding interactions (See details in the text).

It is thought that a key feature of the reversible disassembly of eukaryotic V-ATPases is located on the V_1_–V_o_ interface, involving C_foot_, the distal lobe of a_NT_, and one EG heterodimer (Figure 5A). Gel filtration analysis of an E1G1-a1_NT_-C1 ternary complex revealed a sharp peak with a distinct shoulder (Figure 5B). From the SDS-PAGE profile of the fractions (Figure 5C), we determined that the major peak corresponded to C1-E1G1, and the shoulder corresponded to the unbound a1_NT_ subunit. Both E1G1 and C1 subunits showed low affinity for the a1_NT_ subunit (Table 1), which explains the failure to form a stable ternary complex. By basic native gel electrophoresis, we observed two bands corresponding to E1G1-C1 and unbound a1_NT_ (Figure 5D).

The binding affinity between C1 and a1_NT_ was low (*K_D_* = 3.0 µM) (Table 1). We used SPR to examine the influence of these subunits in forming an E1G1-a1_NT_-C1 ternary complex (Figure 5E). By using high concentrations of analytes (E1G1, 10 µM), both C_head_ and C_foot_ regions of ligand C1 could be activated (Table 1), with a response of 75 RU (+/−5) (Figure 5E, case X). The response of the C1-a1_NT_ interaction was low, 45 RU (+/−5), even in the presence of high concentrations of the analyte (a1_NT_, 10 µM) (Figure 5E, case Y). Finally, we observed a cumulative response, 120 RU (+/−5), by using a mixture of E1G1 and a1_NT_ (10 µM of each) as analytes (Figure 5E, case Z). We interpreted this response to be the sum of their individual responses, attributed to the interaction between their own affinities and the formation of a ternary complex, although the stability of this complex may be very low.

### Investigation of Ternary Interaction in an E1G1-a1_NT_-H Mixture

The H subunit is thought to play an important role in stabilizing the V-ATPase by interacting with the EG heterodimer and the proximal lobe of a_NT_. We expressed and purified a relatively long construct of a1_NT_ (1–353 amino acid residues); therefore, we expected the amino acid residues important for both proximal and distal lobes to be present and properly folded (Figure S7A). The E1G1-a1_NT_-H ternary complex was not detected by gel filtration (Figure S7B); the earlier peak contained H, E1, and G1, and the latter, only unbound a1_NT_ (Figure S7B–C). The affinities between these subunits were not sufficiently strong to form a stable ternary complex (Table 1). In the pulldown assay, His-tagged H subunit interacted only with the non-tagged E1G1 with high affinity (Figure S7D, lane 2), and the a1_NT_ band seemed to be less pronounced as compared with that after elution with buffer B (Figure S7D, lane 1).

### Investigation of Quaternary Interaction in an E1G1-C1-a1_NT_-H Complex

**Figure 6:**
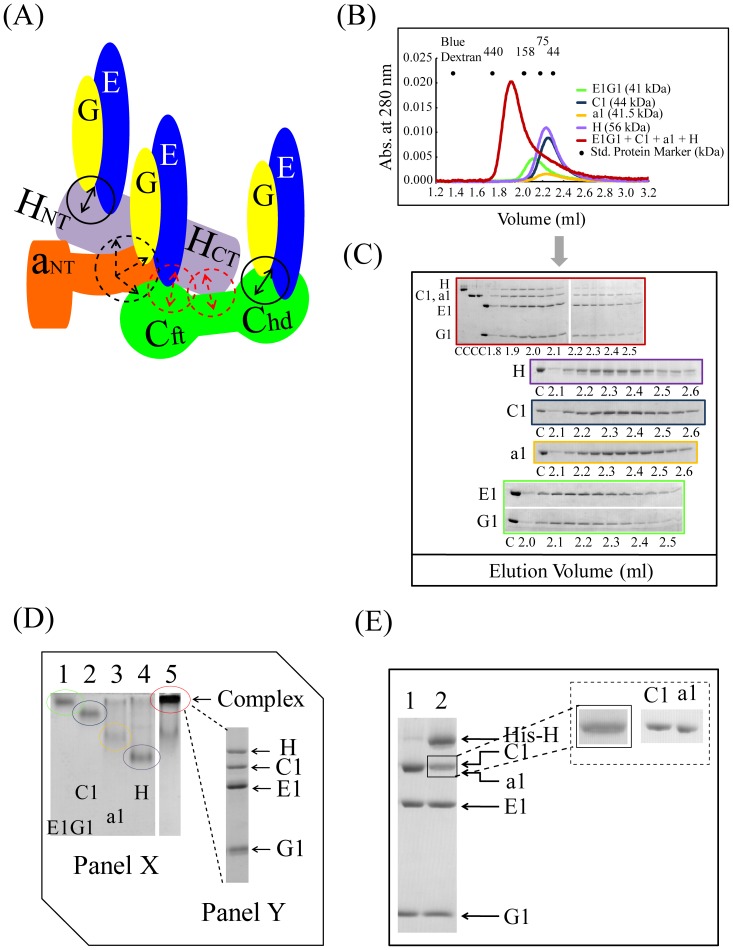
Quaternary interactions of E1G1, C1, a1_NT_, and H. (A) Model of the quaternary subunit assembly. Dotted arrows indicate weak and solid arrows (black) strong binding. (B) Gel filtration profile of H/C1/a1_NT_/E1G1 mixture (red) in comparison to H (purple), C1 (blue), a1_NT_ (yellow), and E1G1 (green) monomers. (C) SDS-PAGE analysis of the eluted fractions from gel filtration chromatography. Border colors indicate samples corresponding to the color scheme used in 6B. “C” indicates control proteins. (D) Panel X: Basic native polyacrylamide gel electrophoresis analysis of the H/C1/a1_NT_/E1G1 mixture. A 3∶2∶1∶1 molar ratio of E1G1:a1_NT_:C1:H proteins was prepared and incubated on ice for 1 h (lane 5). Bands corresponding to one molar amounts of E1G1, C1, a1_NT_, and H proteins are visible in lanes 1, 2, 3, and 4, respectively. Panel Y: SDS-PAGE (12% gel) analysis of the intense band eluted from the native gel in panel X (lane 5), suggesting the presence of C1, E1 and G1 in this complex. An unbound a1_NT_ band was observed at the expected position. (E) SDS-PAGE of the eluted proteins from the His-tag pulldown experiment. Lane 1, fraction eluted using buffer B; lane 2, subunits bound with His-tagged H subunit eluted using buffer C.

**Figure 7:**
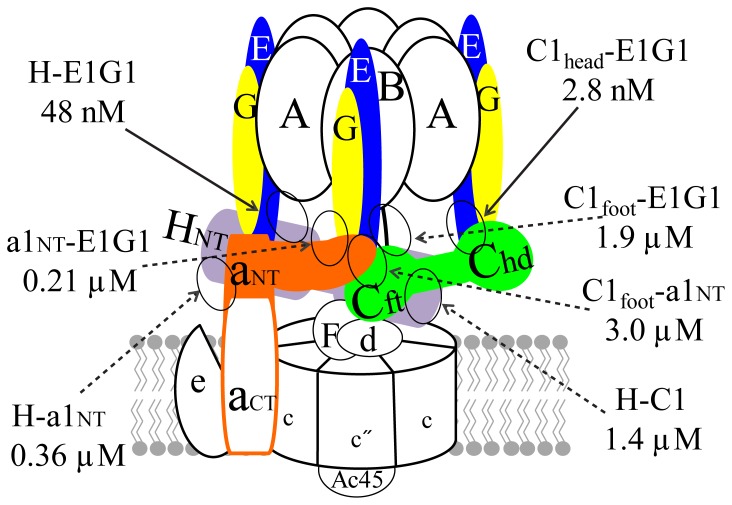
Structural model of human V-ATPase showing *K_D_* values for affinities of subunit-subunit interactions. Diverse affinities between subunits of the peripheral stalk of human V-ATPase are illustrated. Dotted arrows indicate weak and solid arrows strong binding interactions.

A quaternary complex of E1G1, C1, H, and a1_NT_ was not sufficiently stable to be detected by gel filtration (Figure 6A–C); no peak was observed before the position of the other strong complexes (E1G1-C1 or E1G1-H). Almost all of the a1_NT_ appeared at the tail position of the peak, indicating weak affinity of this subunit for the others (Figure 6B). Fractions analyzed by SDS-PAGE showed elution of H, C1, E1, and G1 at 1.85 ml (Figure 6C), with a1_NT_ starting to emerge at approximately 1.95 ml (C1 and a1_NT_ bands could be separated using a 7% gel, data not shown), slightly earlier than a1_NT_ alone. The conditions used for native PAGE were very harsh to identify the weak binding interactions of a1_NT_ with other subunits. The resultant strong band from the mixture of E1G1-C1-a1_NT_-H suggested that only E1G1, C1, and H were part of the complex, with the majority of unbound a1_NT_ observed in its expected migratory position (Figure 6D). However, by pulldown assay, a quaternary complex eluted with the His-tagged H subunit was observed, although the band intensities of C1 and a1_NT_ were relatively low. Bands of C1 and a1_NT_ could be slightly separated, and the intensities of both of these bands were much stronger (Figure 6E, lane 2) than the previous investigation of ternary complex formation (Figure 4E and S7D). Taken together, we speculate that the sum of these individual interactions in the peripheral stalk region (Figure 7) is strong enough to withstand the torque of rotational catalysis and be able to maintain the structural integrity of the complex.

## Discussion

In this study, we investigated the physical interactions between the stator subunits of the human V-ATPase. Subunit interactions were assessed by several biochemical techniques, including gel filtration, native PAGE, pulldown assays, and SPR.

The co-expressed human EG subunit complex was found to be very stable, consistent with findings from *Saccharomyces cerevisiae*, *Enterococcus hirae, Thermus thermophilus,* and *Thermoplasma acidophilum*, suggesting an evolutionarily conserved function for this complex as a stator in all V-ATPases and A-ATPases [22], [28]–[34]. The peak position of E1G1 in the gel filtration assay indicated that the complex may dimerize or oligomerize; however, we do not have any other results than the gel filtration profile for suggesting such higher order complexes and we, therefore, speculate that the observed aberrant elution is a consequence of the elongated structure of the molecule [10], [27]. We observed stable complex formation of E1G1 with both C1 and H subunits, with E1G1C1 and E1G1H complexes being well resolved and eluting as single peak. However, the estimated molecular weights determined from the gel filtration analysis did not correspond well with the calculated values (Figure 2B; Figure 3B); this discrepancy may also be attributed to the elongated or L-shapes of these stator subunits. It was, therefore, not possible to obtain reliable estimations of molecular mass from the gel filtration analysis. *In vivo,* the C subunit of yeast V-ATPase has been shown to interact with two pairs of EG subunits. Our *in vitro* analysis (gel filtration and native PAGE) confirmed a strong interaction between EG and C subunits (possibly via C_head_). However, using the Biacore technique, we obtained two binding affinities of approximately 2.8 nM and 1.9 µM, which may correspond to the C1_head_ and C1_foot_ regions of the C1 subunit, respectively. We also observed a strong interaction between the EG and H subunits.

It is known that C and H subunits are situated at the V_1_–V_o_ junction, forming a base for the three EG heterodimers [8], [10], [35], [36] (Figure 1). Although cryo-electron microscopic studies in yeast have revealed a CE_3_G_3_H assembly structure [27], no biochemical evidence regarding the stability of the ternary binding interaction of C-EG-H subunits has been reported. Therefore, we examined the possibility of ternary complex formation of E1G1, C1, and H subunits by using a 3∶1∶1 E1G1:C1:H molar ratio in gel filtration, native polyacrylamide gel electrophoresis, and pulldown assays (Figure 4). Although by gel filtration, the mixture eluted as a single peak, no peak shift was observed relative to the binary complex positions. When we used equimolar amounts of E1G1, C1 and H (1∶1∶1 molar ratio), we observed the formation of an unstable complex, containing only C1-E1G1 and lacking the H subunit (Figure S6A-B); these results are consistent with our quantitative data showing that the C1_head_-E1G1 complex is more stable than H-E1G1 (Table 1). Our findings suggest that the complex peak observed by gel filtration of the 1∶1∶3 molar ratio of H, C1, and E1G1 mixture (Figure 4B) contains two binary complexes (C1-E1G1 and H-E1G1). The complex did not appear to undergo any conformational changes related to the regulation of the binding activity; the interaction was controlled by the sum of their individual affinities. The weak interaction between subunits C1 and H, even within the E1G1-C1-H ternary complex, indicates the suitability of this bond for the regulatory reversible dissociation of subunit C1 from the V_1_V_o_ complex.

Strong, stable complex formation between C1-H, E1G1-a1_NT_, C1-a1_NT_, and H-a1_NT_ was not observed. However, the position of gel filtration fractions of (E1G1-a1_NT_) indicates that the strong band of a1_NT_ eluted one lane earlier than that of monomer a1_NT_, suggesting a slight influence of interaction between these subunits (). The *K*_D_ values for affinities between the interactions of these subunits were found to be in micromolar range (Table 1). We were unable to estimate the *k_on_* rate and *k_off_* rate for all interactions to evaluate the kinetics of the reaction, and some of the interactions appeared to be complex with unreliable curve fitting due to the background effects of non-specific binding because of the higher concentrations of analytes in these assays. Thus, we estimated the equilibrium *K_D_* from the plots of steady-state analyte binding levels (*R*_eq_) by using several analyte concentrations at equilibrium. Our results suggest that binding affinities between these different subunits at the ternary junction area of V_1_ and V_o_ are low to moderate. No binding influence was observed for the ternary binding interaction; hence, it is likely that no conformational change occurred on ternary complex formation that caused subunit-subunit interaction affinities to change. Thus, the Biacore analysis results suggest that the ternary complex may assemble with avidity, rather than affinity (Figure 5E and 7). Moreover, the weak interactions between these subunits are likely to be crucial for the reversible dissociation initiated at this junction area, which is important for enzyme regulation. By contrast, bacterial A/V-ATPases are not regulated by this type of reversible dissociation and may lack C and H subunits. In bacteria, subunit a_NT_ (NtpI) and the EG complex are known to constitute the stator domain, as reported in *E. hirae* [30] and *T. thermophilus* [37].

We previously reported the expression and purification of the a1_NT_ construct [22]. Although several a1_NT_ subunit constructs were designed, most of these were unstable. Finally, we were able to purify a stable a1_NT_ (1–353) peptide, which appears to fold correctly, because it demonstrated a concentration-dependent binding response with other peripheral stalk subunits in the Biacore experiment. Several interactions observed between a1_NT_ and other soluble subunits provide evidence that this domain is a major point of contact linking the cytoplasmic V_1_ to the membrane domain (V_o_) of V-ATPase [8], [9], [13]. The low-to-moderate affinities of interactions of a1_NT_ with other peripheral stalk subunits observed in this study provide evidence that this model may be consistent for eukaryotic V-ATPase regulation. However, a1_CT_, the membrane domain of the a1 subunit, which protrudes from the proximal lobe, may be crucial for proper functioning of the subunit at this ternary junction point of the peripheral stalk. Model fitting of the N-terminal a subunit with the crystal structure of the N-terminal a subunit of *Meiothermus ruber* revealed that its proximal lobe, located directly below the H subunit, is directed toward the region linking a_NT_ and a_CT_ domains [38]. It is possible that the entire a subunit plays significant role *in vivo* through its interactions with other peripheral stator subunits; however, examination of these functional roles was beyond the scope of our *in vitro* subunit-subunit interaction studies. Moreover, the conformations of the synthesized human proteins may be different from those in the V-ATPase complex. Nevertheless, we were able to demonstrate a moderately more stable quaternary complex formation involving E1G1, C1, H, and a1_NT_. We speculate that a1_NT_ in the quaternary complex was able to weakly interact with E1G1 and the C_foot_ region of the C subunit and that the cumulative strength of these bonds activated additional binding of E1G1 to the C_foot_ region. The results of pulldown assays also suggest that the subunits influence one another in formation of a more stable quaternary complex (Figure 6E). We did not observe any affinity changes due to the cumulative binding, whereas rather high avidity was observed, and this may be sufficiently strong to withstand the torque of rotational catalysis. Hence, multiple variable affinity interactions among stator subunits would be sufficient to allow the overall stator function required to facilitate V-ATPase activity.

This study provides a qualitative and quantitative biochemical analyses of the peripheral stalk subunits of a mammalian V-ATPase. Previous studies conducted on yeast have demonstrated EG-C and EG-H complex formation [25]; however, the kinetics and affinity of the interactions have not been described. Here, we report the binding affinities of the E1G1-C1_head_, E1G1-C1_foot_, and E1G1-H subunit interactions. Association and dissociation rates were estimated to determine the kinetics for the two binding sites of the C1 subunit with regard to E1G1. Recently, the binding affinities between C_foot_-a_NT_ and EG-a_NT_ were determined to be 22 µM and 33 µM, respectively, in yeast, using isothermal titration calorimetry [14]. Here, we reported *K_D_* values of the low affinity interactions between C1-H, E1G1-a1_NT_, C1-a1_NT_, and H-a1_NT_ determined by SPR.

In summary, by characterizing human peripheral stalk subunits utilizing biochemical and biophysical techniques, we demonstrated the strong interactions and ability to resist the torque of rotational catalysis of two stator EG heterodimers with subunits C and H, along with several moderate-to-low affinity multiple intermediary interactions (Figure 7). The weak interactions are likely to be significant in facilitating the disruption of the individual associations during reversible dissociation of the V-ATPase. Specific interactions among these peripheral subunits may thus provide the requisite functional flexibility for enzymatic activity. We speculate that major structural and functional properties of this enzyme are highly conserved among eukaryotes, including vertebrates and invertebrates. However, it is likely that differences exist, depending on the nature of environmental signals and the cellular and subcellular localization of the subunits and isoforms may have a major role in this regard. Our detailed studies on the peripheral stalk subunits of human V-ATPase, revealing the qualitative and quantitative properties, will provide further insights into the function of this enzyme, specifically in mammals. Future work will aim to determine three-dimensional structures from crystals of the stable complexes identified in our study, which may lead identification of drug targets for human diseases caused by deficiencies in subunits of human V-ATPases.

## Supporting Information

Figure S1 **Densitometric analysis of standard proteins for the estimation of binding stoichiometry from the protein amount in gel filtration fraction.** (A) Coomassie blue-stained 12% SDS PAGE profile: Standard amounts of C1 and E1G1 proteins were loaded on lane 1–4 (solid line box) and lane 5–8 (solid line box), respectively. Gel filtration fractions from the mixture of E1G1 and C1 were loaded on lane 9–12 (dotted line box). (B) Standard graph showing the fitted line from the densitometric values of the increasing amounts of C1 and E1G1 standard. The amount of each protein present in the gel filtration fraction was calculated from the linear regression equation of each protein. (C) Coomassie blue-stained 12% SDS PAGE profile: Standard amounts of H and E1G1 proteins were loaded on lane 1–4 (solid line box) and lane 5–8 (solid line box), respectively. Gel filtration fractions from the mixture of E1G1 and H were loaded on lane 9–12 (dotted line box). (D) Standard graph showing the fitted line from the densitometric values of the increasing amounts of H and E1G1 standard. The amount of each protein present in the gel filtration fraction was calculated from the linear regression equation of each protein.
(TIF)

Figure S2 **Interactions between H and C1.** (A) Gel filtration profile of H/C1mixture (red) in comparison with H (purple) and C1 (blue) monomers. (B) SDS-PAGE analysis of the eluted fractions from gel filtration chromatography. Gel border colors indicate samples corresponding to the color scheme used in S2A. “C” indicates control proteins. (C) Real-time binding evaluation was performed using a Biacore system. Sensorgrams for the binding of various concentrations of the analyte (C1) to the ligand (H) are shown. The inset curve shows steady state binding isotherm for binding of C1 at various concentrations to H ligand on a CM5 sensor chip. (D) Basic native polyacrylamide gel electrophoresis analysis of H and C1 interaction. For complex formation, equimolar amounts of H and C1 proteins were mixed and incubated on ice for 1 h (lane 3). Bands corresponding to one molar amount of C1 and H are visible in lanes 1 and 2, respectively. (E) SDS-PAGE of the eluted proteins from the His-tag pulldown experiment. Lane1, fractions eluted using buffer B; lane 2, proteins bound with His-tagged H subunit eluted using buffer C.
(TIF)

Figure S3 **Interactions between E1G1 and a1_NT_.** (A) Gel filtration profile of E1G1/a1_NT_ mixture (red) in comparison to E1G1 (green) and a1_NT_ (yellow) monomers. (B) SDS-PAGE analysis of the eluted fractions from gel filtration chromatography. Gel border colors indicate samples corresponding to the color scheme used in S3A. “C” indicates control proteins. (C) Real-time binding evaluation was performed using a Biacore system. Sensorgrams for the binding of various concentrations of the analyte (E1G1) to the ligand (a1_NT_) are shown. The inset curve shows the steady-state binding isotherm for binding of E1G1 at various concentrations to a1_NT_ ligand on a CM5 sensor chip. (D) Basic native polyacrylamide gel electrophoresis analysis of E1G1 and a1_NT_ interaction. For complex formation, equimolar amounts of E1G1 and a1_NT_ proteins were mixed and incubated on ice for 1 h (lane 3). Bands corresponding to one molar amount of E1G1 and a1_NT_ are visible in lanes 1 and 2, respectively.
(TIF)

Figure S4 **Interactions between C1 and a1_NT_.** (A) Gel filtration profile of C1/a1_NT_ mixture (red) in comparison to C1 (blue) and a1_NT_ (yellow) monomers. (B) SDS-PAGE analysis of the eluted fractions from gel filtration chromatography. Gel border colors indicate samples corresponding to the color scheme used in S4A. “C” indicates control proteins. (C) Real-time binding evaluation was performed using a Biacore system. Sensorgrams for the binding of various concentrations of the analyte (a1_NT_) to the ligand (C1) are shown. The inset curve shows the steady-state binding isotherm for binding of a1_NT_ at various concentrations to C1 ligand on a CM5 sensor chip.
(TIF)

Figure S5 **Interactions between H and a1_NT_.** (A) Gel filtration profile of H/a1_NT_ mixture (red) in comparison to H (purple) and a1_NT_ (yellow) monomers. (B) SDS-PAGE analysis of the eluted fractions from gel filtration chromatography. Gel border colors indicate samples corresponding to the color scheme used in S5A. “C” indicates control proteins. (C) Real-time binding evaluation was performed using a Biacore system. Sensorgrams for the binding of various concentrations of the analyte (a1_NT_) to the ligand (H) are shown. The inset shows the steady-state binding isotherm for binding of a1_NT_ at various concentrations to H ligand on a CM5 sensor chip. (D) SDS-PAGE of the eluted proteins from the His-tag pulldown experiment. Lane1, fractions eluted using buffer B; lane 2, proteins bound with His-tagged H subunit eluted using buffer C.
(TIF)

Figure S6 **Ternary interactions of equimolar amounts of E1G1, C1, and H.** (A) Gel filtration profile of the equimolar amount mixture of H/C1/E1G1(red) in comparison to H (purple), E1G1 (green), and C1 (blue) monomers. (B) SDS-PAGE analysis of the eluted fractions from gel filtration chromatography. Border colors indicate samples corresponding to the color scheme used in S6A.
(TIF)

Figure S7 **Ternary interactions of E1G1, H and a1_NT_.** (A) Possible model of ternary binding interaction of E1G1, H and a1_NT_. Dotted arrows indicate weak and solid arrows (black) strong binding interactions. (B) Gel filtration profile of (E1G1/H/a1_NT_) mixture (red) in comparison to E1G1 (green), H (purple) and a1_NT_ (yellow) monomers. (C) SDS-PAGE analysis of the eluted fractions from gel filtration chromatography. Gel border colors indicate samples corresponding to the color scheme used in S6B. “C” indicates control proteins. (D) SDS-PAGE of the eluted proteins from the His-tag pulldown experiment. Lane1, fractions eluted using buffer B; lane 2, subunits bound with His-tagged H subunit eluted using buffer C.
(TIF)
