# Supplementary figures and images for: Biochemical and Biophysical Properties of Interactions between Subunits of the Peripheral Stalk Region of Human V-ATPase
Source: PLoS One. 2013 Feb 11;8(2):e55704. doi: 10.1371/journal.pone.0055704 (PMC3569449; doi:10.1371/journal.pone.0055704)

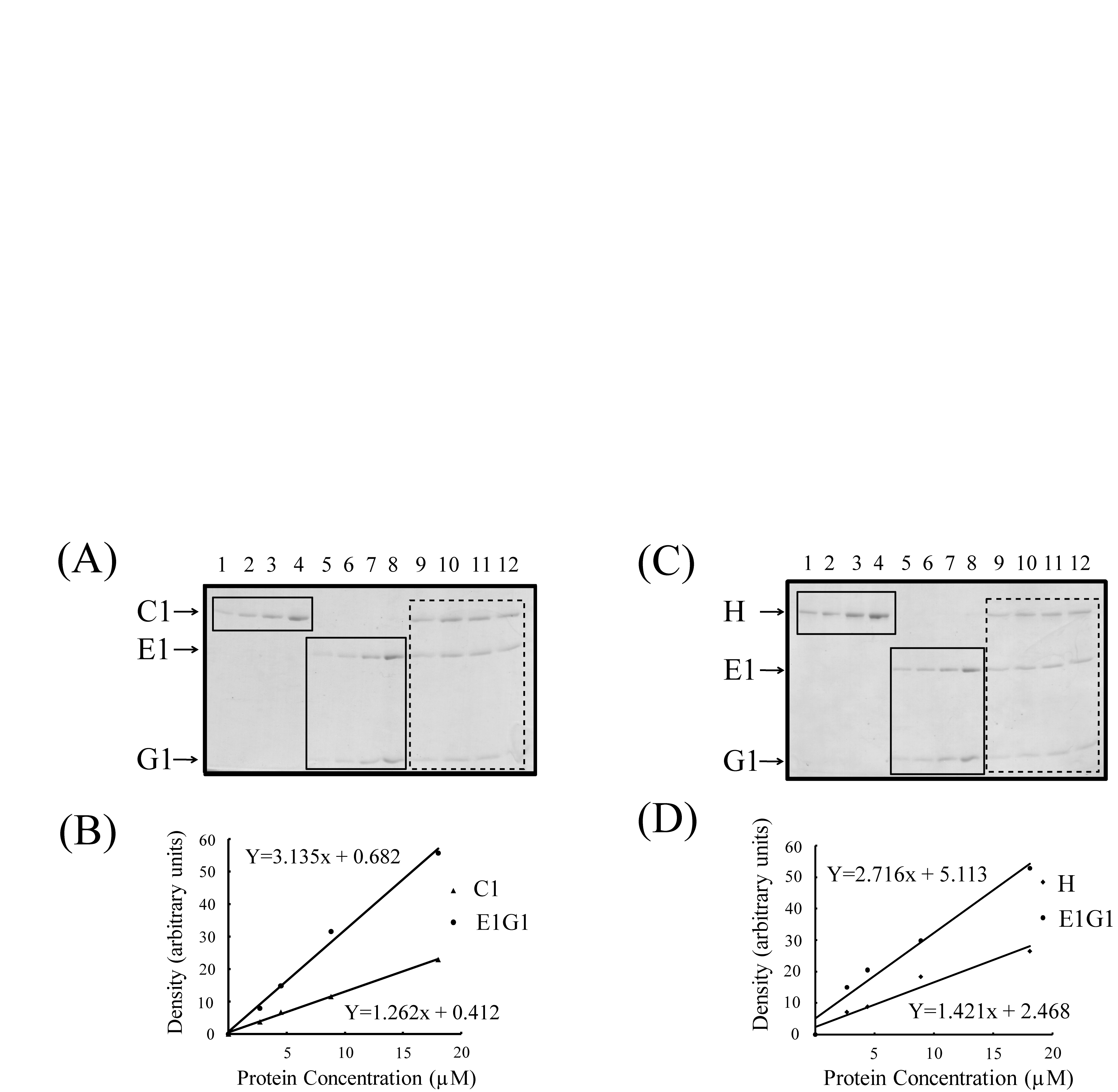

Supplement: Figure S1 — Densitometric analysis of standard proteins for the estimation of binding stoichiometry from the protein amount in gel filtration fraction. (A) Coomassie blue-stained 12% SDS PAGE profile: Standard amounts of C1 and E1G1 proteins were loaded on lane 1–4 (solid line box) and lane 5–8 (solid line box), respectively. Gel filtration fractions from the mixture of E1G1 and C1 were loaded on lane 9–12 (dotted line box). (B) Standard graph showing the fitted line from the densitometric values of the increasing amounts of C1 and E1G1 standard. The amount of each protein present in the gel filtration fraction was calculated from the linear regression equation of each protein. (C) Coomassie blue-stained 12% SDS PAGE profile: Standard amounts of H and E1G1 proteins were loaded on lane 1–4 (solid line box) and lane 5–8 (solid line box), respectively. Gel filtration fractions from the mixture of E1G1 and H were loaded on lane 9–12 (dotted line box). (D) Standard graph showing the fitted line from the densitometric values of the increasing amounts of H and E1G1 standard. The amount of each protein present in the gel filtration fraction was calculated from the linear regression equation of each protein. (TIF) [file pone.0055704.s001.tif]

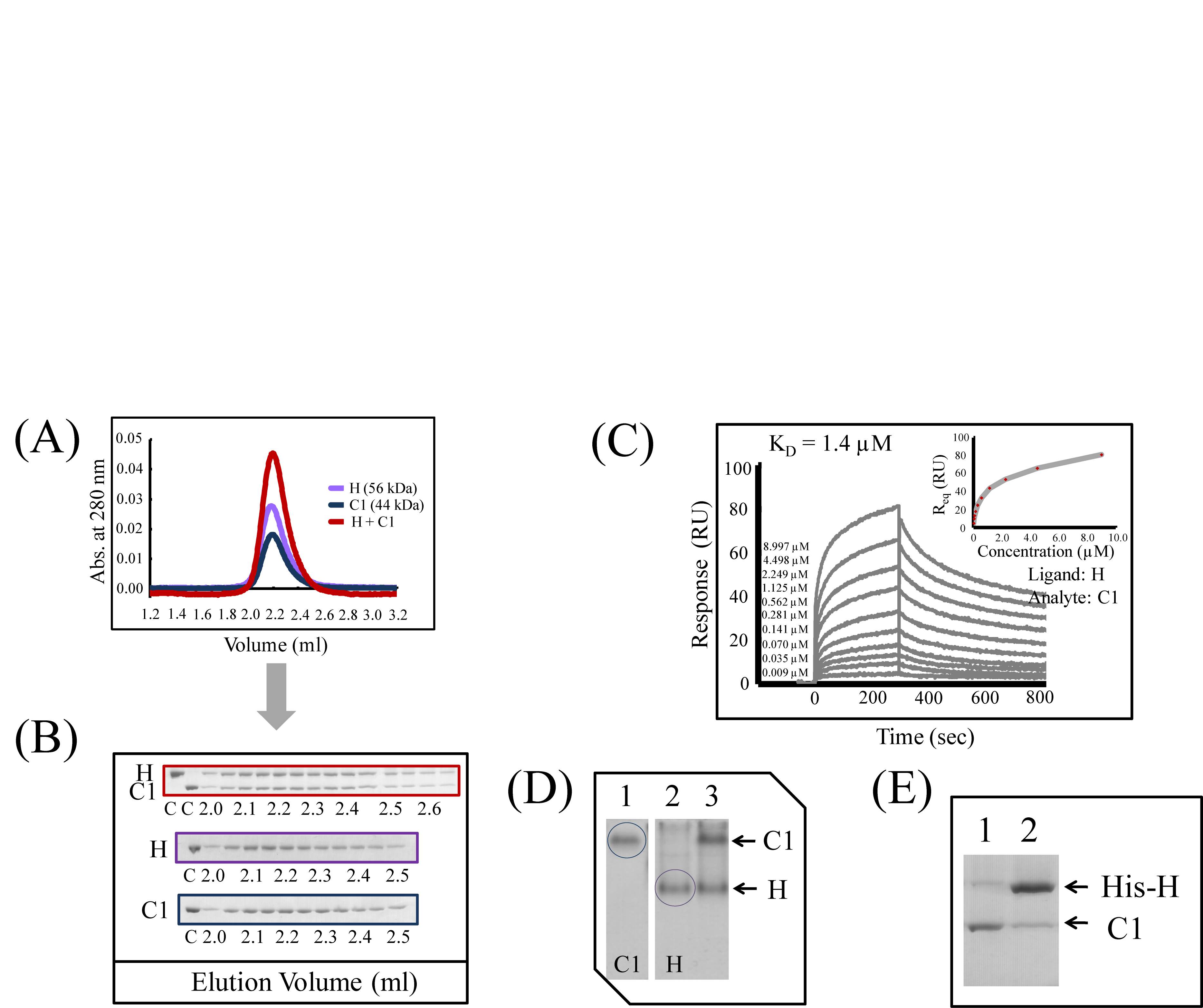

Supplement: Figure S2 — Interactions between H and C1. (A) Gel filtration profile of H/C1mixture (red) in comparison with H (purple) and C1 (blue) monomers. (B) SDS-PAGE analysis of the eluted fractions from gel filtration chromatography. Gel border colors indicate samples corresponding to the color scheme used in S2A. “C” indicates control proteins. (C) Real-time binding evaluation was performed using a Biacore system. Sensorgrams for the binding of various concentrations of the analyte (C1) to the ligand (H) are shown. The inset curve shows steady state binding isotherm for binding of C1 at various concentrations to H ligand on a CM5 sensor chip. (D) Basic native polyacrylamide gel electrophoresis analysis of H and C1 interaction. For complex formation, equimolar amounts of H and C1 proteins were mixed and incubated on ice for 1 h (lane 3). Bands corresponding to one molar amount of C1 and H are visible in lanes 1 and 2, respectively. (E) SDS-PAGE of the eluted proteins from the His-tag pulldown experiment. Lane1, fractions eluted using buffer B; lane 2, proteins bound with His-tagged H subunit eluted using buffer C. (TIF) [file pone.0055704.s002.tif]

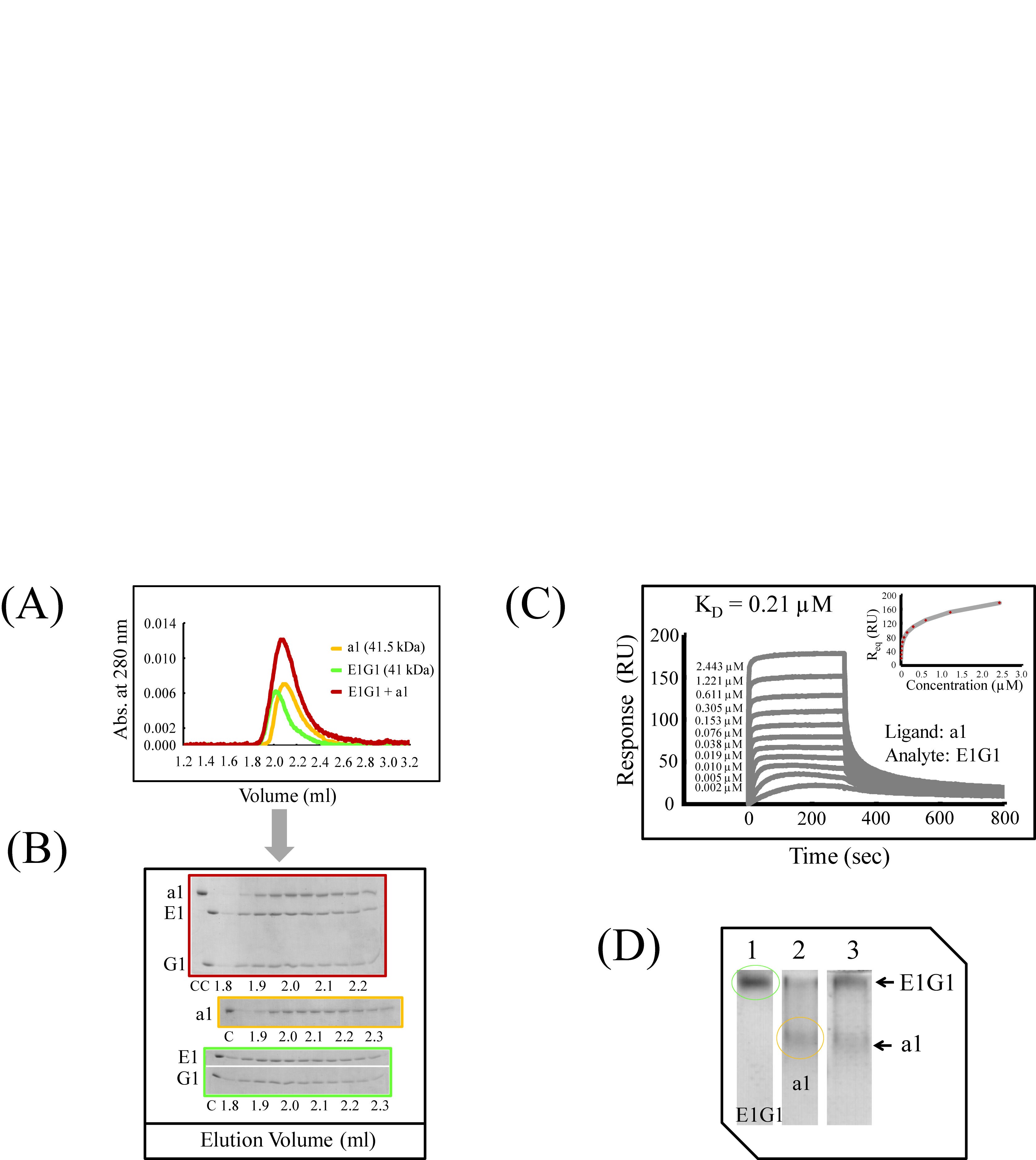

Supplement: Figure S3 — Interactions between E1G1 and a1NT. (A) Gel filtration profile of E1G1/a1NT mixture (red) in comparison to E1G1 (green) and a1NT (yellow) monomers. (B) SDS-PAGE analysis of the eluted fractions from gel filtration chromatography. Gel border colors indicate samples corresponding to the color scheme used in S3A. “C” indicates control proteins. (C) Real-time binding evaluation was performed using a Biacore system. Sensorgrams for the binding of various concentrations of the analyte (E1G1) to the ligand (a1NT) are shown. The inset curve shows the steady-state binding isotherm for binding of E1G1 at various concentrations to a1NT ligand on a CM5 sensor chip. (D) Basic native polyacrylamide gel electrophoresis analysis of E1G1 and a1NT interaction. For complex formation, equimolar amounts of E1G1 and a1NT proteins were mixed and incubated on ice for 1 h (lane 3). Bands corresponding to one molar amount of E1G1 and a1NT are visible in lanes 1 and 2, respectively. (TIF) [file pone.0055704.s003.tif]

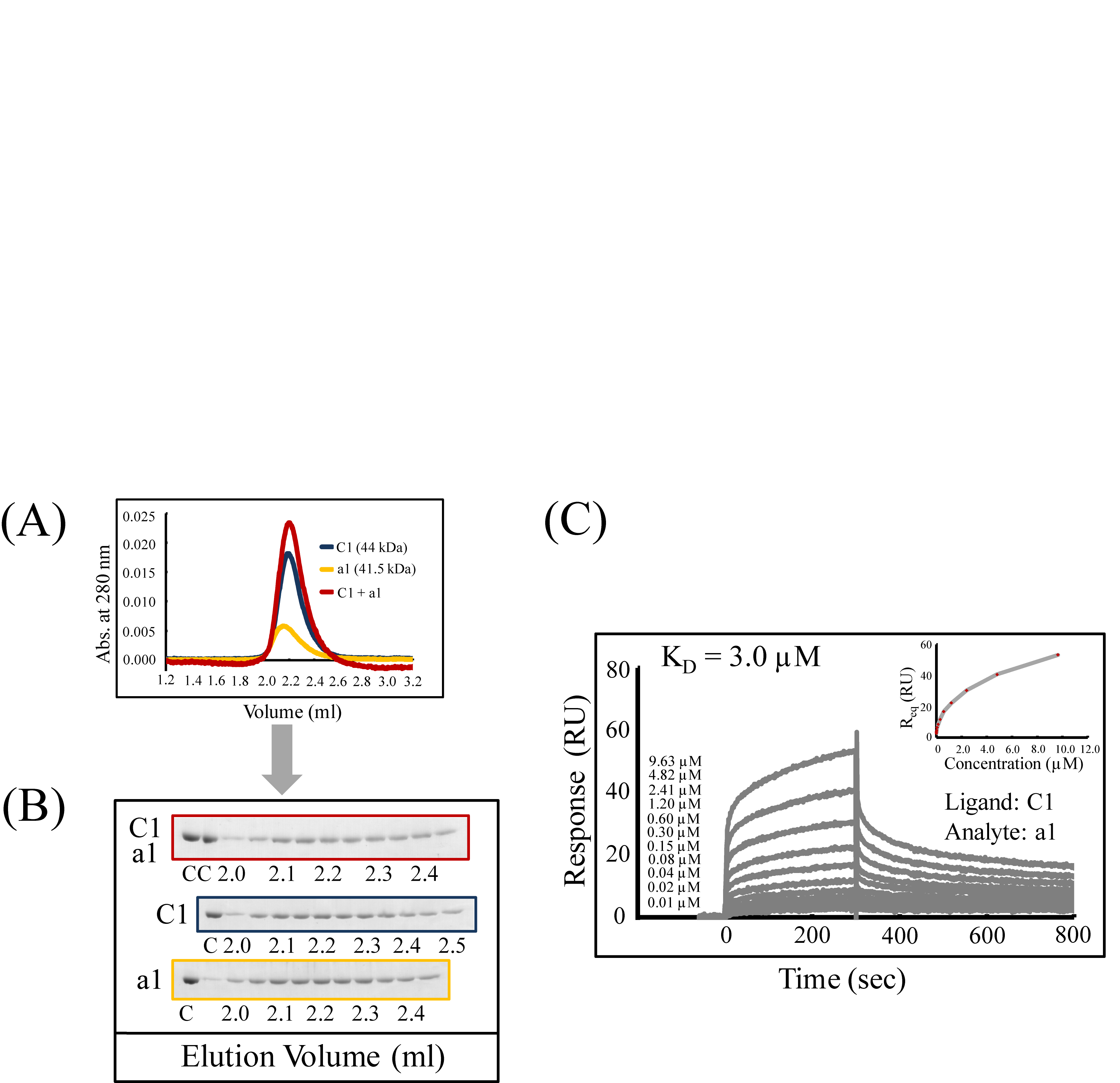

Supplement: Figure S4 — Interactions between C1 and a1NT. (A) Gel filtration profile of C1/a1NT mixture (red) in comparison to C1 (blue) and a1NT (yellow) monomers. (B) SDS-PAGE analysis of the eluted fractions from gel filtration chromatography. Gel border colors indicate samples corresponding to the color scheme used in S4A. “C” indicates control proteins. (C) Real-time binding evaluation was performed using a Biacore system. Sensorgrams for the binding of various concentrations of the analyte (a1NT) to the ligand (C1) are shown. The inset curve shows the steady-state binding isotherm for binding of a1NT at various concentrations to C1 ligand on a CM5 sensor chip. (TIF) [file pone.0055704.s004.tif]

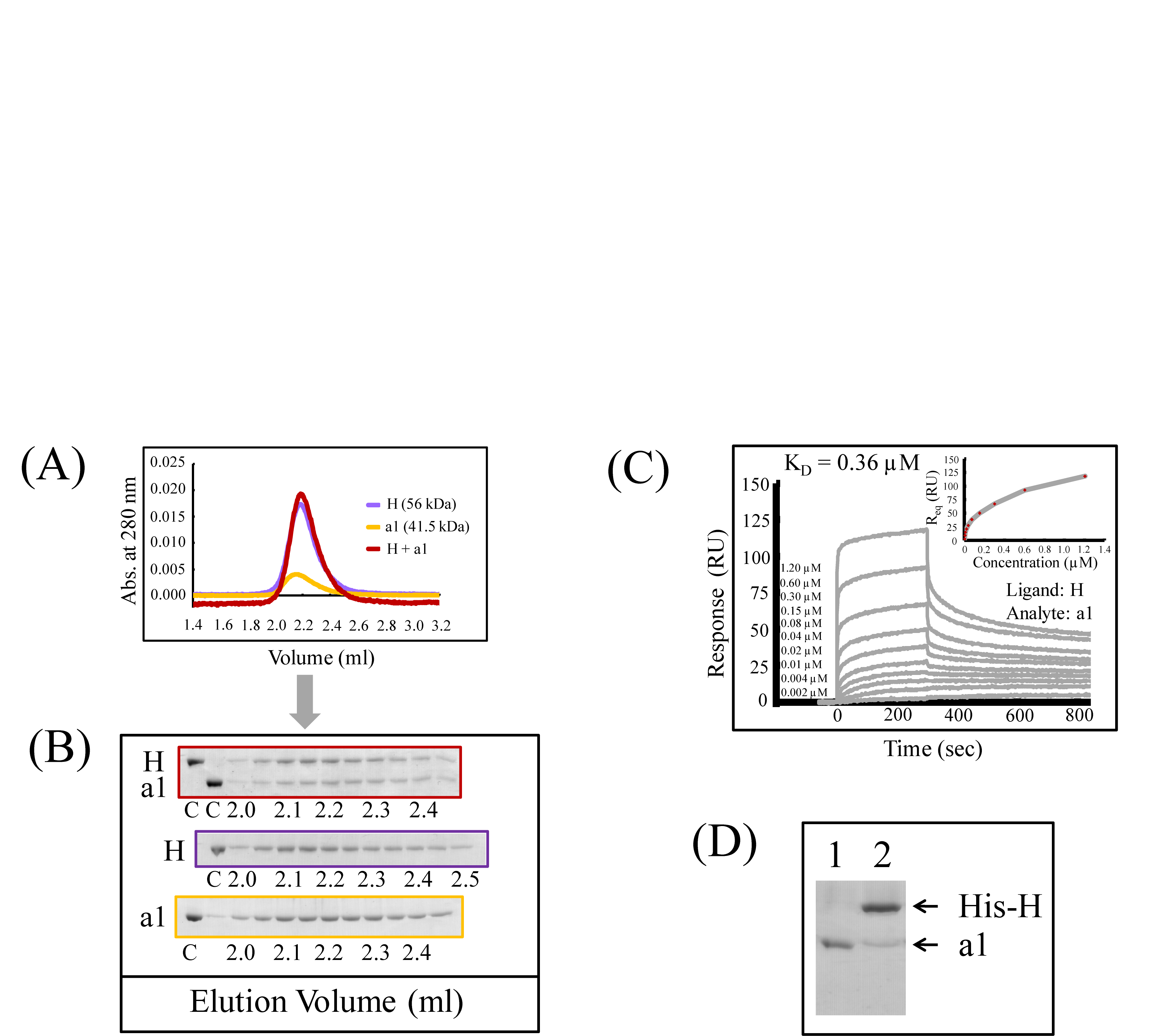

Supplement: Figure S5 — Interactions between H and a1NT. (A) Gel filtration profile of H/a1NT mixture (red) in comparison to H (purple) and a1NT (yellow) monomers. (B) SDS-PAGE analysis of the eluted fractions from gel filtration chromatography. Gel border colors indicate samples corresponding to the color scheme used in S5A. “C” indicates control proteins. (C) Real-time binding evaluation was performed using a Biacore system. Sensorgrams for the binding of various concentrations of the analyte (a1NT) to the ligand (H) are shown. The inset shows the steady-state binding isotherm for binding of a1NT at various concentrations to H ligand on a CM5 sensor chip. (D) SDS-PAGE of the eluted proteins from the His-tag pulldown experiment. Lane1, fractions eluted using buffer B; lane 2, proteins bound with His-tagged H subunit eluted using buffer C. (TIF) [file pone.0055704.s005.tif]

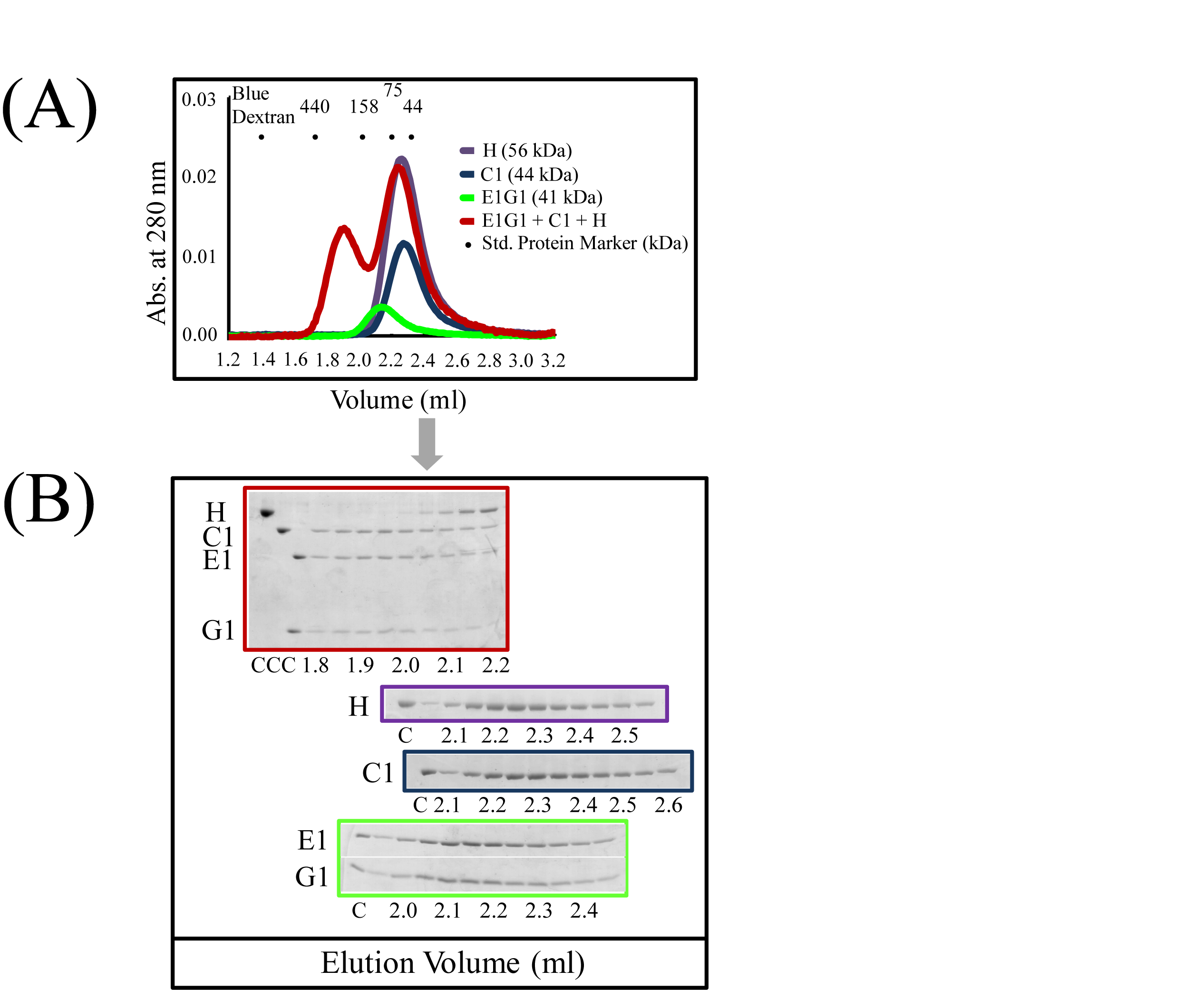

Supplement: Figure S6 — Ternary interactions of equimolar amounts of E1G1, C1, and H. (A) Gel filtration profile of the equimolar amount mixture of H/C1/E1G1(red) in comparison to H (purple), E1G1 (green), and C1 (blue) monomers. (B) SDS-PAGE analysis of the eluted fractions from gel filtration chromatography. Border colors indicate samples corresponding to the color scheme used in S6A. (TIF) [file pone.0055704.s006.tif]

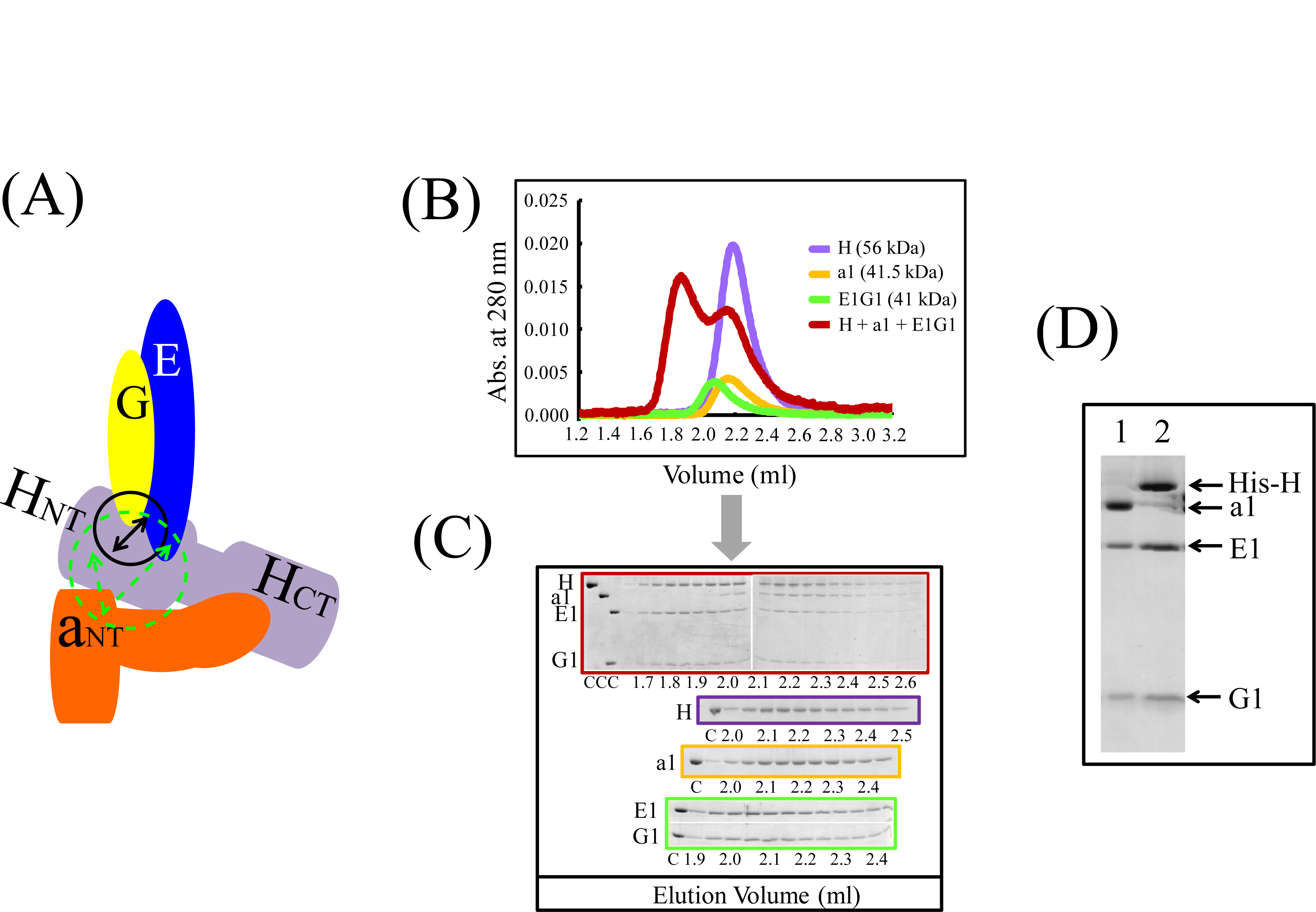

Supplement: Figure S7 — Ternary interactions of E1G1, H and a1NT. (A) Possible model of ternary binding interaction of E1G1, H and a1NT. Dotted arrows indicate weak and solid arrows (black) strong binding interactions. (B) Gel filtration profile of (E1G1/H/a1NT) mixture (red) in comparison to E1G1 (green), H (purple) and a1NT (yellow) monomers. (C) SDS-PAGE analysis of the eluted fractions from gel filtration chromatography. Gel border colors indicate samples corresponding to the color scheme used in S6B. “C” indicates control proteins. (D) SDS-PAGE of the eluted proteins from the His-tag pulldown experiment. Lane1, fractions eluted using buffer B; lane 2, subunits bound with His-tagged H subunit eluted using buffer C. (TIF) [file pone.0055704.s007.tif]
